# Knockdown of KDM5B Leads to DNA Damage and Cell Cycle Arrest in Granulosa Cells via MTF1

**DOI:** 10.3390/cimb45040210

**Published:** 2023-04-07

**Authors:** Yingnan Yang, Yu Cai, Jinjing Guo, Keke Dai, Liang Liu, Zili Chen, Feng Wang, Mingtian Deng

**Affiliations:** Jiangsu Livestock Embryo Engineering Laboratory, Nanjing Agricultural University, Nanjing 210095, China

**Keywords:** KDM5B, granulosa cells, DNA damage, cell cycle

## Abstract

KDM5B is essential for early embryo development, which is under the control of maternal factors in oocytes. Granulosa cells (GCs) play a critical role during oocyte mature. However, the role of KDM5B in GCs remains to be elucidated. In the current study, we found that KDM5B expressed highly in the ovaries and located in goat GCs. Using an RNA sequence, we identified 1353 differentially expressed genes in the KDM5B knockdown GCs, which were mainly enriched in cell cycle, cell division, DNA replication and the cellular oxidative phosphorylation regulation pathway. Moreover, we reported a decrease in the percentage of proliferated cells but an increase in the percentage of apoptotic cells in the KDM5B knockdown GCs. In addition, in the KDM5B knockdown GCs, the percentage of GCs blocked at the S phase was increased compared to the NC group, suggesting a critical role of KDM5B in the cell cycle. Moreover, in the KDM5B knockdown GCs, the reactive oxygen species level, the mitochondrial depolarization ratio, and the expression of intracellular phosphorylated histone H2AX (γH2AX) increased, suggesting that knockdown of KDM5B leads to DNA damage, primarily in the form of DNA double-strand breaks (DSBs). Interestingly, we found a down-regulation of MTF1 in the KDM5B knockdown GCs, and the level of cell proliferation, as well as the cell cycle block in the S phase, was improved. In contrast, in the group with both KDM5B knockdown and MTF1 overexpression, the level of ROS, the expression of γH2AX and the number of DNA DSB sites decreased. Taken together, our results suggest that KDM5B inhibits DNA damage and promotes the cell cycle in GCs, which might occur through the up-regulation of MTF1.

## 1. Introduction

Oocyte maturation is closely related to early embryonic development. Failure of oocyte maturation will seriously affect both female reproductive health and fertility [1]. Increasing studies have shown that abnormal oocyte maturation leads to fertilization failure, embryo development retardation, and embryo death before implantation [2]. Oocyte maturation is achieved with follicular growth and development, during which ovarian granulosa cell (GC) proliferation and apoptosis are required [3,4]. A previous study has revealed that apoptosis of GCs is linked to decreased ovarian reserve in female animals [5]. For example, in polycystic ovary syndrome patients’ granulosa cells, the level of apoptosis and the expression of BAX, CAS3, CAS9 and other vital genes regulating apoptosis increased significantly [6]. Therefore, it is critical to maintain GC proliferation and apoptosis during follicular development.

During proliferation, DNA replication occurs at the S phase of interphase [7]. Every stage of the cell cycle is very important, and the normal entry and development of the S phase is crucial for cell development, tissue repair and immune function [8,9]. After a cell enters mitosis, it is prone to different types of damage, such as DNA double-strand breaks. When DNA DSBs occur during mitosis, the cell will continue to divide without DNA damage repair, so the wrong chromatids are expressed, preventing normal cell division and proliferation [10]. However, overactive or abnormal entry of the S phase can lead to replication stress, DNA damage and canceration [11]. Other studies have shown that the entry of the S phase in the daughter cell G1 phase is independent of mitogen, but this phase is very prone to DNA damage, such as DNA single-strand breaks and DNA double-strand breaks [12]. It was proven that continuous DNA DSBs induced by SPO-11 inhibited *Caenorhabditis elegans* meiosis [13,14]. In granulosa cells, increased DNA DSBs and reduced DNA repair might contribute to ovarian aging [15]. Jose Antonio et al. found that DNA damage caused by environmental influences led the cell cycle to stop. In this way, the damaged DNA was prevented from replicating further [16]. These studies suggest that the occurrence of DNA damage may lead to the obstruction of cell cycle progression, thus leading to abnormal cell division.

It has been reported that reactive oxygen species (ROS) causes adverse changes, such as proliferation arrest, aging, apoptosis and necrosis [17]. Recently, there were research studies pointing out that chloroquine increased ROS in ovarian cancer cell lines, causing DSBs [18,19]. The primary sources of ROS were mitochondria and plasma membrane [20]. ROS oxidizes macromolecules in cells and destroys their normal function, causing cell damage or even cell death [21]. Severe oxidative stress with a higher level of ROS in the ovarian granulosa cells of patients with polycystic ovary syndrome was reported [22]. On the other hand, decreased intracellular ROS levels reduced pancreatic β-cells apoptosis in alloxan-induced larvae [23]. These and subsequent studies suggest that ROS needs to be maintained at a normal level in cells that typically divide and grow [24].

It has been pointed out that epigenetic factors play pivotal roles in DNA DSB repair. For example, histone H3K36me3 methyltransferase SETD2 could promote DNA DSB repair, thus maintaining genome stability [20]. KDM5B (also known as JARID1B/PLU1), a H3K4me2/3 histone demethylase, is also indispensable for DNA damage responses. Previous studies have demonstrated that attenuation of KDM5B alters H3K4 methylation patterns, affects DNA damage responses, increases DNA DSBs and reduces DNA damage repair ability [25]. Studies have shown that KDM5B leads to ROS overproduction by regulating SIRT3, thereby leading to oxidative stress and mitochondrial metabolism disorder [26]. Moreover, KDM5B is thought to modulate the expression of tumor-suppressing genes and oncogenes, thus affecting cancer cell proliferation [27,28]. Further research has revealed that the knockdown of KDM5B significantly inhibits the proliferation of hepatocellular carcinoma cells both in vivo and in vitro by arresting the cell cycle at the G1/S phase, partly by up-regulating P15 and P27 [29,30]. However, the function of KDM5B in GCs remained to be elucidated.

MTF1 is a metal transcriptional regulator, which encodes a transcription factor that induces the expression of metallothionein and other genes involved in metal homeostasis in response to heavy metals, such as cadmium, zinc, copper and silver [31]. It has been reported that the accumulation of zinc and copper ions induced by MTF1 leads to cell apoptosis [32]. However, to our best knowledge, there is no research pointing out the effects of KDM5B and MTF1 in goat GCs. Additionally, no research has focused on the regulatory relationship between KDM5B and MTF1.

Using small interfering RNA, RNA sequencing and rescue experiments, we investigated the expression pattern and function of KDM5B in goat granulosa cells in the present study. We found that knockdown of KDM5B caused DNA damage and S phase arrest in GCs. In contrast, overexpression of MTF1 reduced DNA DSBs and S phase arrest, suggesting that knockdown of KDM5B caused DNA damage and S phase arrest in GCs by repressing MTF1.

## 2. Materials and Methods

### 2.1. Sample Collection

All animal experiments were performed according to the Measures for the Administration of Laboratory Animals of Nanjing Agricultural University and approved by the Animal Care and Use Committee of Nanjing Agricultural University. Goat heart, liver, spleen, lung and kidney were cut into small pieces and stored in liquid nitrogen. Parts of the ovaries were immediately frozen at −196 °C for subsequent experiments. In contrast, the remaining parts of the ovaries were fixed in 4% formaldehyde for at least 24 h and embedded in paraffin for immunofluorescence staining.

### 2.2. Granulosa Cell Culture

During the breeding seasons, goat ovaries were collected from the Danyang abattoir (Zhenjiang, Jiangsu, China) (October to December). GCs were isolated from healthy follicles (2–5 mm) and subsequently cultured with DMEM/F12 glutaMAX (Gibco, Thermo Fisher Scientific, Waltham, MA, USA) and 10% FBS (Certified Foetal Bovine Serum, Biological Industries, Kibbutz Beit-Haemek, Israel, 25115) at 37 °C with 5% CO_2_. When the cells reached 80–90 percent growth in the flask, they were frozen at −196 °C for further study.

### 2.3. Transfection of siRNA against KDM5B

GCs were thawed and cultured in T25 (TC Flask T25, 5 × 10^6^ cells/flask) before being passed into 6-well plates (2 × 10^6^ cells/well). GCs were only transfected once after thawing to ensure greater cell viability, and no other passages were permitted. At 60–70% confluence, GCs were transfected with siRNA against KDM5B (Genepharma, Shanghai, China), which was premixed with lipofectamine 3000 transfection reagent (Invitrogen, Waltham, MA, USA) according to the manufacturer’s instructions. A well of cells was transfected with the universal negative control siRNA to exclude the non-specific influence of the siRNA treatment (NC). The sequences of siRNAs are shown in Table 1. The transfected cells were harvested for RNA extraction after 48 h of transfection and for protein extraction after 72 h of transfection.

### 2.4. Quantitative Real-Time PCR

Trizol reagent (Invitrogen, CA, USA) was used to extract total RNAs from the goat tissues and GCs. Reverse transcription was performed using a Strand cDNA Synthesis Kit with a gDNA wiper (Vazyme Biotech Co., Ltd., Nanjing, China). Following the manufacturer’s instructions, all the qPCR reactions were performed in a QuantStudio 5 Real-Time PCR system (Applied Biosystems, Foster City, CA, USA) using Chamq Universal SYBR qPCR Master Mix (Vazyme Biotech Co., Ltd., Nanjing, China). The reaction was performed as follows: annealing at 95 °C for 30 s, followed by 40 cycles at 95 °C for 10 s and at 60 °C for 30 s, and, finally, a dissociation step consisting of 95 °C for 15 s, 60 °C for 60 s, and 95 °C for 15 s. The 2^−ΔΔCT^ method was used to quantify relative mRNA expression, and β-actin (ACTB) was used as an internal control. All experiments were repeated at least three times independently. The primer sequences are listed in Table 2.

### 2.5. Immunofluorescence Staining

Immunofluorescence was performed as described in a previous study [33]. Briefly, the transfected GCs were passaged into a 24-well plate on round coverslips, and they were fixed with 4% paraformaldehyde (PFA) for 15 min at room temperature (RT). Then, the cells were permeabilized using 0.5% Triton X-100 in DPBS for 20 min at RT and blocked in 5% BSA in DPBS for one hour at RT, followed by incubation with anti-KDM5B/PLU1/JaridlB antibody (1:500, Abcam, Waltham, MA, USA) at 4 °C overnight. After washing in DPBS, the cells were incubated for one hour at RT with Alexa fluor goat anti-rabbit IgG (Boster, 1:1000) in the dark. Then, the cells were washed with DPBS three times. The nuclei were stained with Hoechst 33342 for 5 min at RT. Before the coverslips were placed on a glass slide, an antifade mounting medium was dripped on the glass slide. Then, a laser scanning confocal microscope (Carl Zeiss, Oberkochen, Germany) was used to observe the cells with the same condition.

### 2.6. Cell Proliferation Analysis

Cell proliferation was assessed using the Alexa Fluor 647-Click-iT EdU Assay Kit (KGA332-50, Beyotime Biotechnology, Shanghai, China). The transfected goat GCs were cultured in a 24-well plate on round coverslips and incubated with 100 mM EdU for 2 h. Subsequently, the cells were fixed with 4% PFA for 20 min at RT and washed with 2 mg/mL of glycine for 5 min. The cells were then permeabilized in DPBS containing 0.5% Triton X-100 for 20 min. After washing with DPBS, the nuclei were stained with Hoechst 33342 for 5 min at RT. Before the coverslips were placed on a glass slide, an antifade mounting medium was dripped on the glass slide. Finally, a laser scanning confocal microscope was used to observe the cells with the same condition.

### 2.7. Flow Cytometry Analysis

The cell culture fluid of the transfected GCs was collected, and the cells in the 6-well plate were digested with trypsin for 3 min. Then, the culture fluid was added to the solution to terminate the digestion. The cell suspensions were obtained twice, mixed and centrifuged at 1200–1500 r/min. After washing with DPBS, the cell suspension was centrifuged again. Finally, the single-cell suspension was mixed with DPBS and then sent to Nanjing Liangwei Biotechnology Co., Ltd. (Nanjing, China) immediately for flow cytometry analysis to detect cell apoptosis. We calculated the proportion of cells with early and later apoptosis.

The cell culture fluid of the transfected GCs was discarded, and then the cells were digested, washed and centrifuged to make a single-cell suspension with 70% alcohol and sent to Wuhan Service Co., Ltd. (Wuhan, China) for flow cytometry analysis to detect the cell cycle stage.

### 2.8. Western Blot Analysis

Western blot was performed as described in a previous study [33]. The goat tissues and the transfected GCs were harvested and lysed using RIPA buffer with a protease inhibitor (Thermo Pierce, Waltham, MA, USA). After 20 min of incubation on ice, the ultrasonic cell disruptor was used to disrupt the cells in order to lyse them completely. Next, protein concentrations were measured using a BCA protein assay kit (Beyotime, China). Protein was denatured using Nupage sample reduction agent (10×) and Nupage LDS sample buffer (5×) from Thermo Pierce in the United States. The supernatant was combined with the abovementioned reagents and boiled for 10 min at 70 °C. Then, 20 μg of protein was loaded onto a 12% SDS-polyacrylamide gel and transferred onto polyvinylidene fluoride (PVDF) membranes from each group (Millipore; Billerica, MA, USA). After blocking the membrane with 5% BSA for 2 h at room temperature, it was incubated overnight at 4 °C with primary antibodies (diluted according to Table 3). As the internal controls, β-actin (ACTB) was used. The membranes were washed with TBST for a minimum of one hour, followed by one-hour incubation with secondary antibodies (diluted per Table 3). Immunoblotting was visualized with enhanced ECL ultra-sensitive luminescence fluid (Thermo Pierce, USA) and exposed with Image Quant LAS 400. (Fiji film, Tokyo, Japan). The Image J 1.x (National Institutes of Health, Bethesda, MD, USA) software was used to analyze the changes in protein levels.

### 2.9. Intracellular Reactive Oxygen Species Detection

The ROS Assay Kit (S0033S, Beyotime Biotechnology) was used to assess ROS in the GCs. The cell culture medium was removed, and 1 mL of DCFH-DA diluted 1:1000 in a serum-free medium was added. Then, the cells were incubated for 20 min in a 37 °C incubator. Serum-free DMEM/F12 glutaMAX was used for washing the cells three times. Lastly, a laser scanning confocal microscope (Carl Zeiss, Oberkochen, Germany) was used to observe the cells. ROS was detected using an excitation light of 488 nm and an emission light of 525 nm.

### 2.10. Mitochondrial Membrane Potential Detection

JC-1 (KGMP021, KeyGEN Biotechnology, Nanjing, China) was used to detect mitochondrial membrane potential in the GCs. The JC-1 working solution was prepared according to the instructions of KGMP021 and added to a 6-well plate. Then, the cells were incubated for 20 min in a 37 °C incubator. Lastly, a laser scanning confocal microscope (Carl Zeiss, Oberkochen, Germany) was used to observe the cells. JC-1 monomers were detected using an excitation light of 460 nm and an emission light of 530 nm, and JC-1 multimers were detected using an excitation light of 525 nm and an emission light of 500 nm.

### 2.11. RNA Sequence Data Analysis

RNA libraries were constructed using the Smart-seq2 method. Then, cDNA was fragmented by dsDNA fragments (M0348S, NEB) by incubating at 37 °C for 30 min, and size selection was performed with the provided sample purification beads. The fragmented cDNA at 150–300 bp was used for library construction. This was followed by paired-end sequencing on an illumine Novaseq 6000 platform (LC bio, Hangzhou, China). Quality control was performed to remove adaptors and low-quality bases using fastp (v0.19.6). All reads that passed quality control were mapped to the goat genome ARS1 using HISAT2 (v2.2.1) with the default settings. Uniquely mapped reads were subsequently assembled into transcripts guided by the reference annotation using featureCounts (v2.0.1). Differential expression analysis was performed using DESeq2 (v3.11). Genes with log2 (fold change) >1 or <−1 and with *p*-value < 0.05 were deemed as differentially expressed genes (DEGs).

### 2.12. GO, KEGG and GSEA Analysis

The DEGs were annotated using the Gene Ontology (GO) database (http://www.geneontology.org/, accessed on 20 May 2022), and hypergeometric tests examined these genes’ biological functions and pathways. *p*-values were calculated, and GO terms were considered significantly enriched when *p* < 0.01. The enriched pathways were analyzed using the Kyoto Encyclopedia of Genes and Genomes (KEGG) database (http://www.kegg.jp, accessed on 20 May 2022), and pathways with *p* < 0.01 were considered significantly enriched pathways. The R (v4.2, New Zealand) programming language, including the Biocoductor software packages (v3.17, New Zealand), was mainly used for statistical analysis and data visualization. Heatmap and boxplot of coding potential score, volcano, gene expression, GO, and signal intensity were generated using R package pheatmap (v1.0.12, New Zealand) and ggplot2 (v3.3.2, New Zealand), respectively. The Gene Set Enrichment Analysis (GSEA) of all DEGs was performed using the cluster Profiler (v4.0, New Zealand) and enrichplot R package (v1.10.2, New Zealand). For detailed analysis, please see Deng et al.’s article [33].

### 2.13. Statistical Analysis

All experiments were repeated at least three times, and the data are presented as means ± standard error of the mean based on three independent experiments. Statistical analyses were performed with GraphPad Prism 8 (GraphPad, San Diego, CA, USA) using the independent sample student’s *t*-test. *p*-values < 0.05 were considered as indicating statistically significant differences. A *p*-value < 0.01 indicates a more significant difference than a *p*-value < 0.05.

## 3. Results

### 3.1. High Expression of KDM5B in Goat GCs

**Figure 1:**
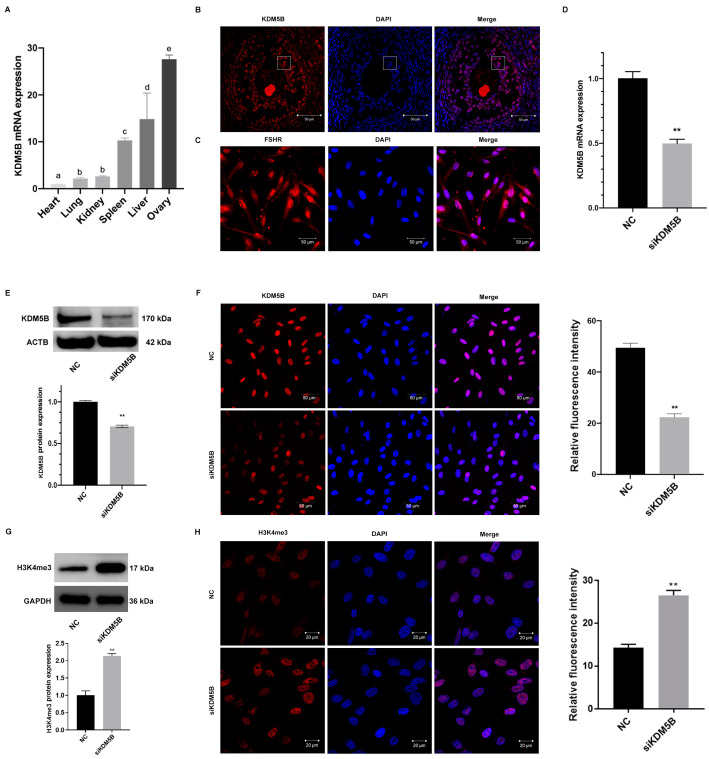
High expression and successful knockdown of KDM5B in goat GCs. (**A**) mRNA expression of KDM5B shows the highest level in goat ovary. Different letters (a–e) represent significant differences between groups, the same letter represents no significant differences between groups. (**B**) Immunofluorescence for KDM5B in goat ovary. KDM5B is located in goat GCs. The squares indicate granulosa cells. (**C**) FSHR is expressed in goat GCs specifically. (**D**) mRNA expression of KDM5B decreases significantly in KDM5B knockdown goat GCs relative to the NC group. (**E**) KDM5B protein level decreases significantly in goat GCs treated with siKDM5B compared to the NC group, as determined by Western blot. (**F**) Immunofluorescence with KDM5B antibody proves that the protein level of KDM5B decreases significantly. (**G**) H3K4me3 level increases significantly in goat GCs treated with siKDM5B compared to the NC group, as determined by Western blot. (**H**) Immunofluorescence with H3K4me3 antibody proves that the H3K4me3 level increases significantly. ** means *p* < 0.01.

To investigate the expression profile of KDM5B, we performed qPCR. As shown in Figure 1A, KDM5B is highly expressed in the ovary compared to the heart, liver, spleen, lung, and kidney tissues in goats. Furthermore, the immunofluorescence staining reveals that KDM5B is localized in GCs (Figure 1B), suggesting that KDM5B might play an important role during follicular development. We therefore isolated GCs from goat ovarian follicle and found a high expression of FSHR, one of the markers of GCs, suggesting that GCs were successfully established (Figure 1C).

### 3.2. Knockdown of KDM5B in Goat GCs

We next transfected siRNAs against KDM5B into the goat GCs to knockdown the expression of KDM5B. As expected, the mRNA expression of KDM5B decreases significantly in the KDM5B knockdown GCs compared to the controls (*p* < 0.01; Figure 1D). Furthermore, the protein level of KDM5B also significantly decreases as revealed by Western blot and immunofluorescence staining (*p* < 0.01; Figure 1E,F). As KDM5B demethylates H3K4me2/3, we explored changes in the H3K4me3 methylation levels. As shown in Figure 1G,H, H3K4me3 methylation levels increase significantly in the KDM5B knockdown GCs compared to the NC group, as revealed by Western blot and immunofluorescence staining. These data suggest that the expression of KDM5B has been successfully knocked down.

### 3.3. Compromise of Cell Cycle and DNA Replication-Related Functions in KDM5B Knockdown GCs

**Figure 2:**
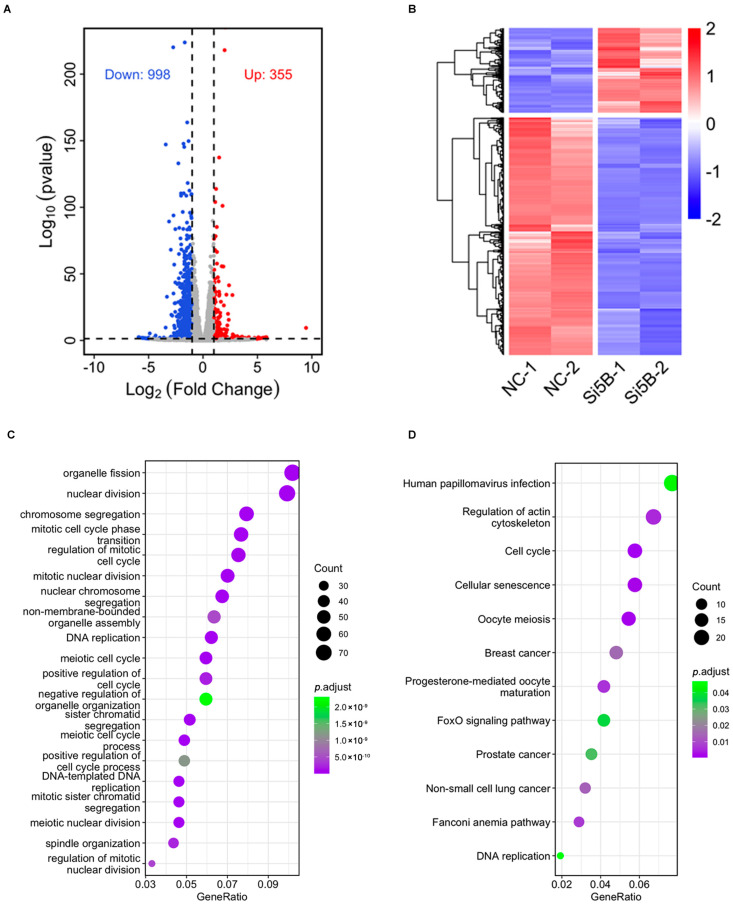
GO and KEGG functional enrichment analysis. (**A**) Volcanic plots display that a total of 1353 DEGs have been identified, of which 998 genes are down−regulated and 355 genes are up−regulated. (**B**) Heat map displays that the repeatability within the group is good and the difference between the groups is large. (**C**) Significantly enriched GO terms. (**D**) Significantly enriched KEGG terms.

To determine the gene expression profiles in the KDM5B knockdown GCs, we performed RNA sequencing. Using DESeq2, we identified 1353 differentially expressed genes (DEGs). Of which, 998 genes are down-regulated, while 355 genes are up-regulated, in the KDM5B knockdown GCs compared to the NC group (Figure 2A,B). It is important to know the pathway that genes are de-repressed and down-regulated in knockdown experiments. As shown in Figure 2C, the down-regulated DEGs are mainly enriched in cell cycle, cell division, DNA replication and chromosome segregation. The KEGG analysis reveals that the down-regulated genes are enriched in cell cycle regulation, oocyte meiosis, DNA replication, and the cellular oxidative phosphorylation regulation pathway (Figure 2D). These data suggest that KDM5B might play an important role in cell cycle processing, DNA replication and oxidative phosphorylation.

### 3.4. Blocked S Phase and Restrained Proliferation in KDM5B Knockdown GCs

**Figure 3:**
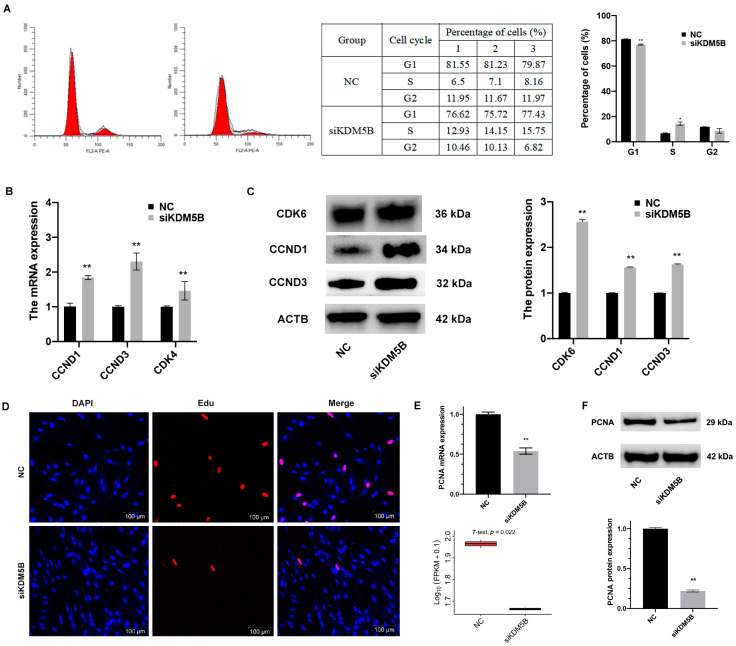
Blocked S phase and restrained proliferation in KDM5B knockdown GCs. (**A**) Flow cytometry results show that the percentage of cells in the G1 phase significantly decreases in the KDM5B knockdown goat GCs and the percentage of cells in the S phase increases compared to the NC. Red represents cells in G1 and G2 phases. (**B**) mRNA expressions of Ccnd1, Ccnd3 and CDK4 increase in the KDM5B knockdown goat GCs compared to the NC, as determined by qPCR. (**C**) Western blot shows that CDK6, Ccnd1 and Ccnd3 protein levels increase in the KDM5B knockdown goat GCs compared to the NC. (**D**) Goat GCs treated with siKDM5B show decreased Edu incorporation relative to the NC. Hoechst indicates nuclear staining. The scale bars correspond to 100 μm. (**E**) mRNA expression of PCNA significantly decreases in the KDM5B knockdown goat GCs relative to the NC. (**F**) Western blot shows that PCNA protein level significantly decreases in the KDM5B knockdown goat GCs relative to the NC. ** means *p* < 0.01, * means 0.01 < *p* < 0.05.

The GSEA revealed the DEGs were enriched in cell cycle (*p* < 0.01; Figure 3A). We therefore examined cell cycle distribution after the knockdown of KDM5B to investigate the effect of KDM5B knockdown in cell cycle processing. In the KDM5B knockdown GCs, the percentage of cells in the G1 phase significantly decreases (*p* < 0.01), while the percentage of cells in the S phase significantly increases (*p* < 0.05) when compared to the NC group (Figure 3B). Meanwhile, the mRNA expressions of cell cycle-related genes, such as Ccnd1 (*p* < 0.01), Ccnd3 (*p* < 0.01) and CDK4 (*p* < 0.01), are significantly increased in the KDM5B knockdown GCs compared to the NC group (Figure 3C). Moreover, the protein levels of CDK6 (*p* < 0.01), Ccnd1 (*p* < 0.01) and Ccnd3 (*p* < 0.01) are all significantly increased (Figure 3D) in the KDM5B knockdown GCs. These results and our RNA-seq data indicate that the knockdown of KDM5B leads to S phase block in GCs.

Then, we explored the proliferation of GCs when KDM5B was knocked down. As shown in Figure 3E, the percentage of proliferated cells significantly decreases (*p* < 0.01) in the KDM5B knockdown GCs compared to the NC group, suggesting the knockdown of KDM5B inhibits the proliferation of GCs in goat. To further confirm the result, we investigated the expression of marker genes of proliferation. As shown in Figure 3F, the expression of PCNA significantly decreases in the KDM5B knockdown GCs compared to the controls (*p* < 0.01). Consistently, the protein expression of PCNA also significantly decreases when KDM5B has been knocked down (*p* < 0.01; Figure 3F). Our data suggesting that the knockdown of KDM5B blocks the cell cycle to S phase and restrains proliferation in goat GCs.

### 3.5. DNA DSBs in KDM5B Knockdown GCs

**Figure 4:**
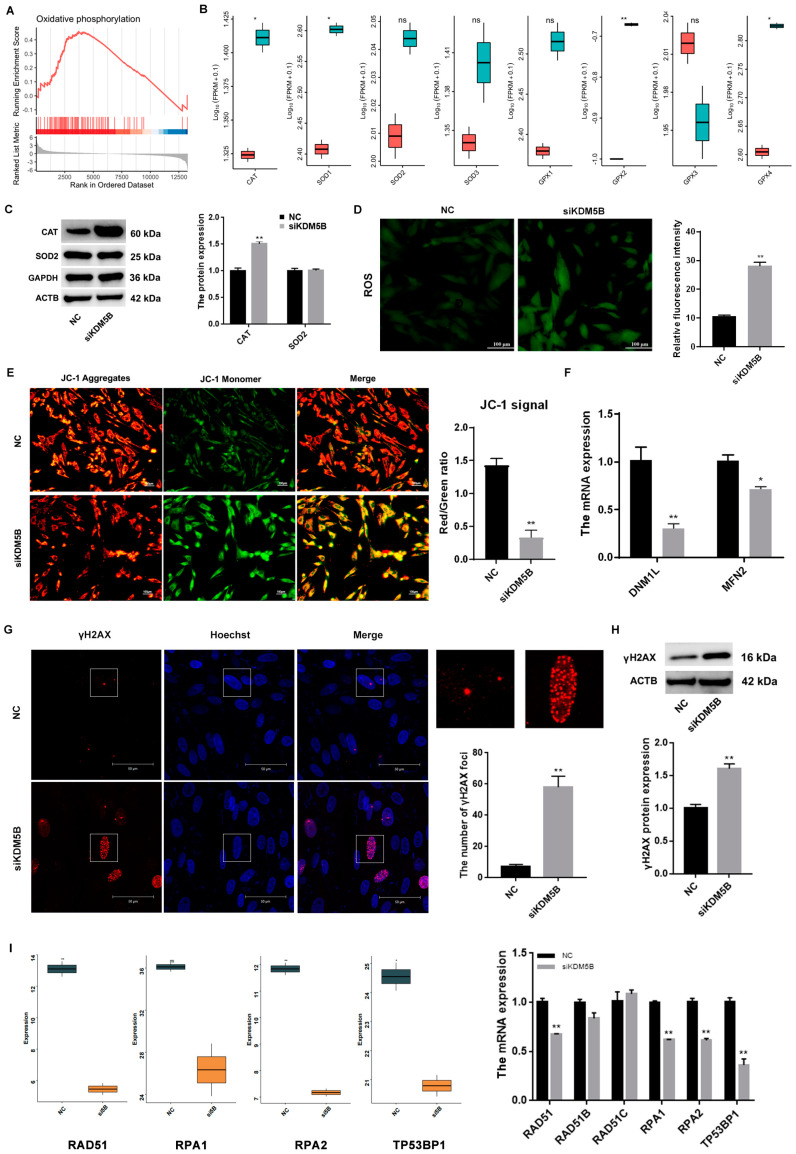
Increased oxidative stress level and DNA damage in KDM5B knockdown GCs. (**A**) Enriched KEGG items revealed by GSEA. DEGs indicate differentially expressed genes. (**B**) mRNA expressions of CAT, SOD1, GPX2 and GPX4 significantly increase. SOD2, SOD3 and GPX4 show an increasing trend, but it is not significant. (**C**) CAT protein level increases, but there is no difference in SOD2 in the goat GCs treated with siKDM5B compared to the NC, as determined by Western blot. (**D**) ROS level increases significantly after the knockdown of KDM5B. (**E**) The results indicate that JC−1 aggregates, as indicated by red fluorescence, significantly decrease, and JC−1 monomers, as indicated by green fluorescence, significantly increase. The ratio of red/green also significantly decreases. (**F**) mRNA expressions of DNM1L and MFN2 significantly decrease. (**G**) γH2AX foci are increased significantly in the KDM5B knockdown group. The white squares represent a typical DNA DSBs site. (**H**) γH2AX protein level increases significantly after the knockdown of KDM5B. (**I**) mRNA expressions of RAD511, RPA1, RPA2 and TP53BP1 significantly decrease after the knockdown of KDM5B. * means *p* < 0.05, and ** means *p* < 0.01. ns means the difference is not significant.

The GSEA reveals the DEGs are enriched in oxidative phosphorylation (*p* < 0.01; Figure 4A). Moreover, genes that regulate oxidative stress, such as CAT, SOD1, GPX2 and GPX4, are elevated significantly after the knockdown of KDM5B (Figure 4B). Consistently, the protein expression of CAT increases significantly (*p* < 0.01; Figure 4C). Therefore, we examined changes in ROS after the knockdown of KDM5B. As shown in Figure 4D, ROS fluorescence intensity is significantly increased in the KDM5B knockdown GCs (*p* < 0.01), indicating that the intracellular ROS level has distinctly increased. Meanwhile, the ratio of red/green is also significantly decreased (*p* < 0.01; Figure 4E), and the mRNA expressions of DNM1L (*p* < 0.01) and MFN2 (*p* < 0.05), which are related to mitochondrial function, both decrease significantly (Figure 4F). γH2AX refers to phosphorylated H2AX, which is a specific indicator for the detection of DNA damage. A large amount of histone H2AX phosphorylates at the DNA DSB sites and forms a fluorescent-resolved focal point [34]. With the knockdown of KDM5B, the number of DNA damage sites and the expression of γH2AX increase significantly compared to the NC group (*p* < 0.01; Figure 4G,H). Meanwhile, the mRNA expressions of RAD511, RPA1, RPA2 and TP53BP1 significantly decrease after the knockdown of KDM5B (*p* < 0.01; Figure 4I). Our data suggest that KDM5B knockdown results in DNA damage by increasing ROS, the proportion of mitochondrial depolarization, and DNA DSBs in GCs.

### 3.6. Promotion of Apoptosis in KDM5B Knockdown GCs

**Figure 5:**
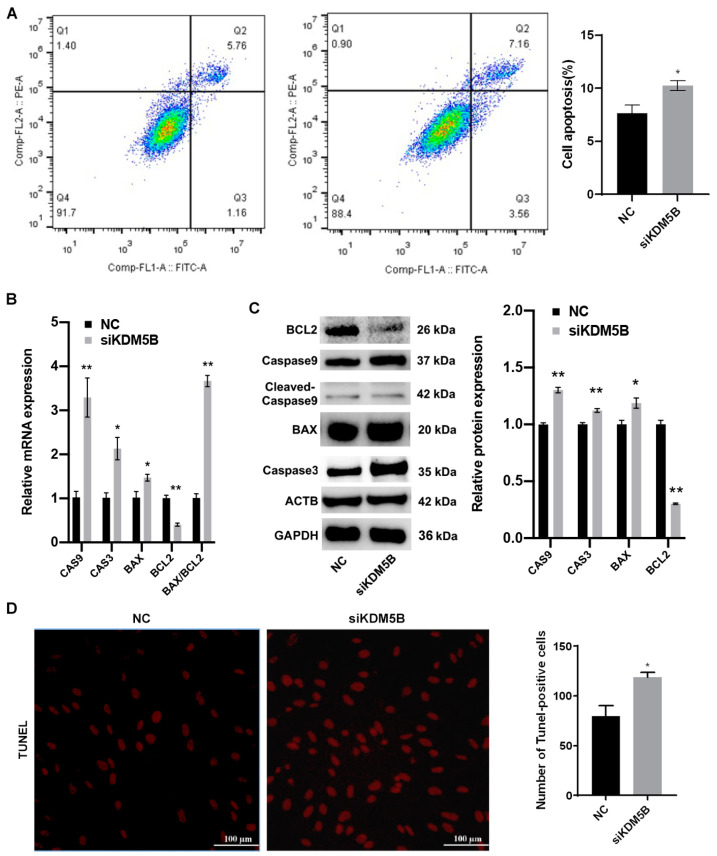
Promotion of apoptosis in KDM5B knockdown GCs. (**A**) Flow cytometry results show that the percentage of apoptotic cells in the goat GCs treated with siKDM5B increases compared to the NC. Q3 represents healthy living cells, Q4 represents early apoptotic cells, Q2 represents late apoptotic cells, and Q1 represents unprogrammed death cells. (**B**) mRNA expressions of CAS9, CAS3 and BAX and the ratio of BAX/BCL2 significantly increase in the KDM5B knockdown goat GCs, while mRNA expression of BCL2 decreases compared to the NC. (**C**) CAS9, CAS3 and BAX protein levels increase, while BCL2 protein level decreases in the goat GCs treated with siKDM5B compared to the NC, as determined by Western blot. (**D**) The result of the tunel assay shows that apoptotic cells are significantly increased in the KDM5B knockdown GCs. * means *p* < 0.05, and ** means *p* < 0.01.

Elevated levels of ROS lead to cell apoptosis. As shown in Figure 5A, the percentage of apoptotic cells significantly increases in the KDM5B knockdown group compared to the NC group (*p* < 0.01; Figure 5A). Moreover, the mRNA expressions of the pro-apoptotic factors, BAX, CAS3 and CAS9, increase significantly, whereas the expression of the anti-apoptotic factor BCL2 decreases, in the KDM5B knockdown GCs (*p* < 0.01; Figure 5B). Additionally, the ratio of BAX/BCL2 significantly increases (*p* < 0.01; Figure 5B). Meanwhile, the protein expressions of BAX, CAS3 and CAS9 increase significantly, whereas the protein expression of BCL2 decreases in the KDM5B knockdown GCs (*p* < 0.01; Figure 5C). The result of TUNEL also intuitively indicates that apoptotic cells are significantly increased in the KDM5B knockdown GCs (Figure 5D). These data suggest that the knockdown of KDM5B accelerates apoptosis in goat GCs.

### 3.7. Rescued Cell Proliferation and Cell Cycle in KDM5B Knockdown and MTF1 Overexpression GCs

**Figure 6:**
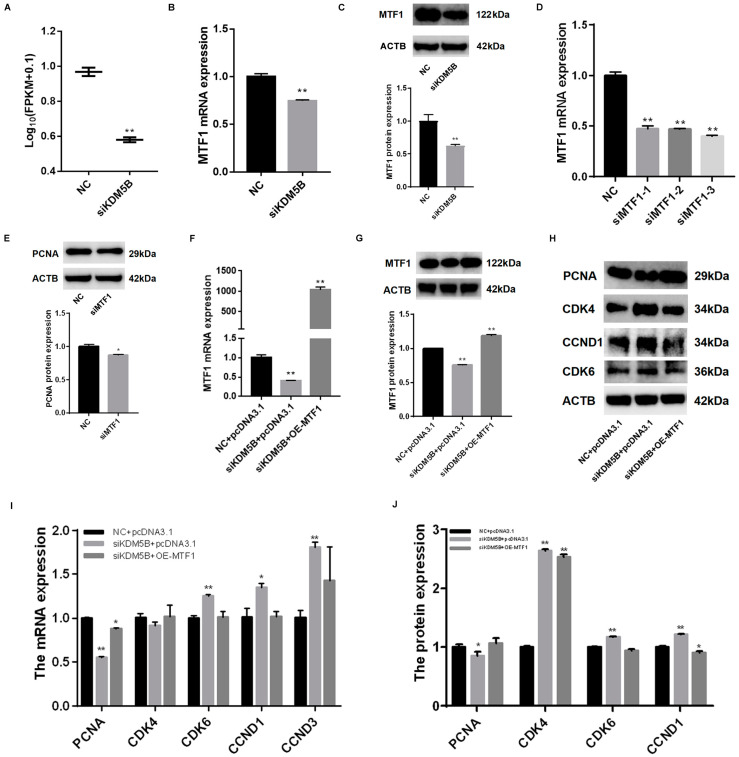
KDM5B maintains normal cell proliferation and cell cycle in GCs via MTF1. (**A**,**B**) mRNA expression of MTF1 significantly decreases after the knockdown of KDM5B. (**C**) Protein expression of MTF1 significantly decreases after the knockdown of KDM5B. (**D**) Knockdown efficiency result shows that siMTF1-3 is the most efficient. (**E**) Protein expression of PCNA significantly decreases after the knockdown of MTF1. (**F**,**G**) mRNA expression of MTF1 significantly decreases after the knockdown of KDM5B, and mRNA and protein expressions of MTF1 significantly increase after the simultaneous knockdown of KDM5B and overexpression of MTF1. (**H**–**J**) mRNA and protein expressions of PCNA decrease significantly and rebound after the simultaneous knockdown of KDM5B and overexpression of MTF1. mRNA and protein expressions of CDK4, CDK6, CCDN1 and CCND3 increase in the KDM5B knockdown GCs and fall back after the simultaneous knockdown of KDM5B and overexpression of MTF1. * means *p* < 0.05, and ** means *p* < 0.01.

MTF1 is one of the differentially expressed genes after the knockdown of KDM5B, as revealed by RNA-seq, qPCR and Western blot (*p* < 0.01; Figure 6A–C). To investigate the role of MTF1, we knocked down MTF1 using siRNA (*p* < 0.01; Figure 6D) and found that the expression of PCNA significantly reduces (*p* < 0.01; Figure 6E). Furthermore, the mRNA and protein expressions of MTF1 are successfully rescued after the knockdown of KDM5B and overexpression of MTF1 in GCs (Figure 6F,G). Interestingly, we found that the mRNA expression (*p* < 0.01) and protein level (*p* < 0.05) of PCNA increase significantly in the KDM5B knockdown and MTF1 overexpression GCs (Figure 6H,I). The mRNA expressions of CDK6 (*p* < 0.01), CCDN1 (*p* < 0.05) and CCND3 (*p* < 0.01) decrease after the simultaneous knockdown of KDM5B and overexpression of MTF1 (Figure 6H). The protein expressions of CDK4 (*p* < 0.01), CCDN1 (*p* < 0.01) and CDK6 (*p* < 0.01) decrease after the simultaneous knockdown of KDM5B and overexpression of MTF1 (Figure 6I). These data suggest that KDM5B might regulate cell proliferation and cell cycle via MTF1, which needs further investigation.

### 3.8. Decreased DNA Damage in KDM5B Knockdown and MTF1 Overexpression GCs

**Figure 7:**
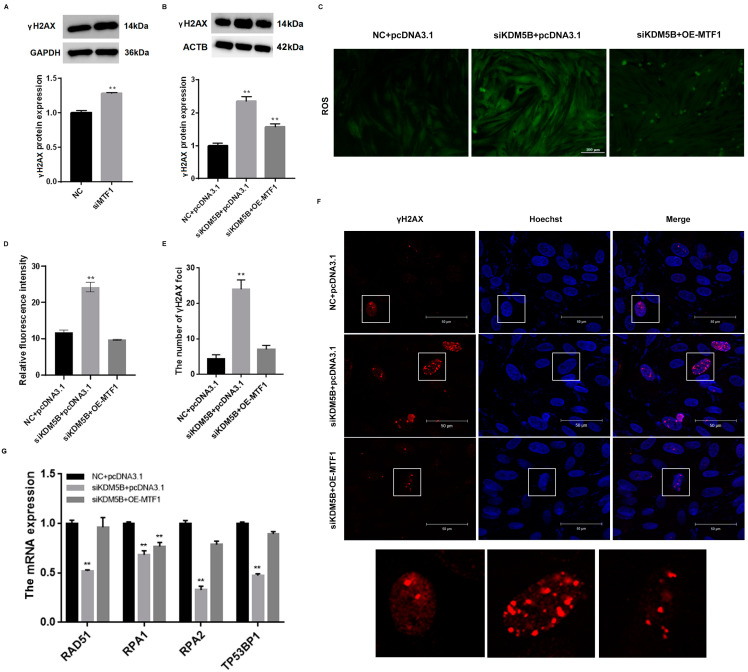
KDM5B inhibits DNA damage in GCs via MTF1. (**A**) Protein expression of γH2AX significantly increases after the knockdown of MTF1. (**B**) Protein expression of γH2AX increases significantly in the KDM5B knockdown GCs and falls back after the simultaneous knockdown of KDM5B and overexpression of MTF1. (**C**,**D**) ROS level increases in the KDM5B knockdown GCs and falls back after the simultaneous knockdown of KDM5B and overexpression of MTF1. (**E**,**F**) γH2AX immunofluorescence results show increasing DNA DSB sites in the KDM5B knockdown GCs, and DNA DSB sites decrease significantly after the simultaneous knockdown of KDM5B and overexpression of MTF1. (**G**) mRNA expressions of RAD511, RPA1, RPA2 and TP53BP1 significantly decrease after the knockdown of KDM5B and fall back after the simultaneous knockdown of KDM5B and overexpression of MTF1. ** means *p* < 0.01.

The expression of γH2AX significantly increases after the knockdown of MTF1 (*p* < 0.01; Figure 7A). Through the rescue experiments, we validated that the expression of γH2AX increases significantly (*p* < 0.01) and decreases after the simultaneous knockdown of KDM5B and overexpression of MTF1 (Figure 7B). Then, we detected the level of ROS, and the results indicate that the fluorescence intensity of the knockdown KDM5B group is significantly higher than control group (*p* < 0.01) and decreases after the simultaneous knockdown of KDM5B and overexpression of MTF1 (Figure 7C,D). The results of the IF intuitively show that DNA damage sites and fluorescence intensity of γH2AX significantly increase after the knockdown of KDM5B (*p* < 0.01). DNA damage sites and fluorescence intensity of γH2AX significantly reduce after the simultaneous knockdown of KDM5B and overexpression of MTF1 (Figure 7E,F). The mRNA expression of RAD511, RPA1, RPA2 and TP53BP1 significantly decrease after the knockdown of KDM5B and fall back after the simultaneous knockdown of KDM5B and overexpression of MTF1 (Figure 7G).

## 4. Discussion

KDM5B is essential for cell proliferation, cell fate and genome stability [35]. However, its function in goat GCs remains unclear. In the present study, we found that knockdown of KDM5B led to an increase in DNA DSBs, cell apoptosis and cell cycle arrest. Additionally, we inferred that KDM5B affected cell cycle and DNA damage via MTF1. Our findings laid the groundwork for future research into the role of KDM5B in follicular development.

KDM5B is highly expressed in the ovary of goats and plays a vital role in DNA DSBs. Increased intracellular ROS levels result in DNA DSBs, affecting DNA replication and cell proliferation [36]. Increased reactive oxygen species in ovarian cancer cells have been found to cause DNA DSBs [37]. Previous research has demonstrated that attenuation of KDM5B altered H3K4 methylation patterns during the genome activation phase of early pig embryos, disrupted embryonic genome activation, altered DNA damage responses, increased DNA DSBs and decreased DNA damage repair capacity, which corresponded to the outcomes of our experiment [24,38]. Our results indicated that the knockdown of KDM5B led to an increase in ROS levels and DNA DSB sites, indicating that DNA damage indeed occurred. It was further confirmed that the sequencing results were affected by the reliability of DNA replication-related functions. We also demonstrated that these DNA DSBs were caused by MTF1 by simultaneously inhibiting KDM5B and overexpressing MTF1. Similar to previous findings, MTF1 can affect intracellular ROS levels by regulating zinc redox, thereby resulting in cell damage [39]. Through the detection of DNA damage repair marker genes, we found that after the KDM5B knockdown, the expressions of RAD511, RPA1, RPA2 and TP53BP1 significantly decreased. These results mean that knockdown of KDM5B leads to reduced DNA damage repair ability and decreased DNA damage repair level. However, this damage was repaired after the overexpression of MTF1. Severe oxidative stress and increased mitochondrial depolarization both lead to cell apoptosis [40,41,42]. Previous research has indicated that KDM5B plays an essential role in the apoptosis of cancer cells [43]. Yang et al. found that LET-7i could target KDM5B to induce apoptosis in esophageal cancer cells [44]. Our study proved that KDM5B accelerated GCs apoptosis. Combined with the previous results, we speculated that this apoptosis was caused by increased intracellular ROS and the occurrence of DNA DSBs.

DNA DSBs are an essential factor affecting DNA replication [45,46]. Because the S phase is the phase of DNA replication, we suspect that cell cycle progression is also affected after the knockdown of KDM5B. Wang et al. demonstrated that KDM5B is frequently up-regulated in HCC specimens and cell lines. Knockdown of KDM5B significantly inhibits cellular proliferation of HCC cells and arrests cell cycle progression at the G1 phase [47]. The present study revealed that the knockdown of KDM5B blocked cell division in the S phase by regulating relative genes. The cell cycle phases arrested in our experiments were different from previous experiments. We hypothesized that the effect mechanism of KDM5B on the cell cycle in GCs was different from that in HCCs. Our experiments also confirmed that KDM5B affected the cell cycle in goat GCs. The elevation of related genes caused many cells to turn from the G1 phase to the S phase. DNA DSBs hindered DNA replication and the cell cycle was arrested in the S phase [48,49]. Through the rescue experiments, we could speculate that the effect of KDM5B on the cell cycle was achieved through MTF1.

The cell cycle determines the fate of cell proliferation to some extent [50,51]. Previous research showed that KDM5B blocked the proliferation of gastric cancer cells. It dramatically promoted gastric cancer cell xenograft formation and growth and promoted gastric cancer cell metastasis in a liver metastasis model [52]. Our results also demonstrated that the knockdown of KDM5B induced a decrease in cell proliferation in goat GCs. In conclusion, the knockdown of KDM5B affected cell cycle progress, standard division and proliferation. Our results provide new insights into the functional mechanisms of KDM5B in granulosa cells.

We found that KDM5B affects MTF1 expression to ensure normal cell proliferation and cell cycle, maintain DNA replication and reduce DNA damage, primarily in DNA DSBs. However, the mechanism of KDM5B and MTF1 interaction remains unknown. We intend to use CHIP experiments in the future to confirm how KDM5B affects MTF1 expression. This way, the mechanism of KDM5B on GCs’ cell cycle arrest and DNA damage will be better understood.

## 5. Conclusions

In conclusion, our results suggest that KDM5B plays a crucial role in goat GCs, as evidenced by its ability to induce DNA DSBs, block the S phase of the cell cycle, restrain cell proliferation and hasten cell apoptosis when it is knocked down. Through the simultaneous knockdown and overexpression of KDM5B and MTF1, we discovered that KDM5B induces DNA DSBs and arrests the cell cycle. These findings have laid the groundwork for future research into the effect and influence of KDM5B on GCs and follicular development. Additionally, this study provides a new reference for the regulatory relationship between KDM5B and MTF1.

## Figures and Tables

**Table 1:** Details of siRNA sequences used for cell transfection.

Genes	Sense (5′-3′)
Negative control (NC)	S:5′-UUCUCCGAACGUGUCACGUTT-3′ A:5′-ACGUGACACGUUCGGAGAATT-3′
KDM5B-goat	S:5′-GCGACUAGUGAGCACUAUUTT-3′ A:5′-AAUAGUGCUCACUAGUCGCTT-3′
MTF1-goat	S:5′-CUCUGUCCUAACUAGUAAUTT-3′ A:5′-AUUACUAGUUAGGACAGAGTT-3′

**Table 2:** Primer sequences, predicted product size of genes, and accession number for RT-qPCR.

Gene	Primer Sequence (5′-3′)	Product Size (bp)	Accession Number
KDM5B	F 5′-TCCTTGAGATGGCAGCACTC-3′ R 5′-CCTTCTGACACACGCAGACT-3′	134	XM_018060847.1
PCNA	F 5′-TGGCTCCCAAGATCGAGGAT-3′ R 5′-TAAAACTGCATTTAGAGTCAAGACC-3′	381	NM_002592.2
CASP3	F 5′-TGAAACATGCCGCCTTCCTA-3′ R 5′-AGTGGCATACCCACATGACTG-3′	242	XM_018041755.1
CASP9	F 5′-CCAGATGCCGTGTCTAGTCTG-3′ R 5′-ACAGTAAGGTAGGGTGAGGGG-3′	296	XM_005690814.3
BAX	F 5′-GCATCCACCAAGAAGCTGAG-3′ R 5′-CCGCCACTCGGAAAAAGAC-3′	130	XM_002701934.1
BCL2	F 5′-ATGTGTGTGGAGAGCGTCA-3′ R 5′-AGAGACAGCCAGGAGAAATC-3′	182	NM_001166486.1
CDK4	F 5′-CCTTCATGCCAACTGCATCG-3′ R 5′-GGCAGTCCGATCAGGTCAAA-3′	309	XM_005680266.3
Ccnd1	F 5′-AGACCCTCGCTTGTGCTTAC-3′ R 5′-AACGTGCCGGTTACATGTCT-3′	117	XM_018043271.1
Ccne3	F 5′-GGATGGAGCTGCTGTGCTG-3′ R 5′-ACAGCTTCTCGATGGTCAGG-3′	375	XM_018038733.1
3β-HSD	F 5′-AGACCAGAAGTTCGGGAGGAA-3′ R 5′-TCTCCCTGTAGGAGTTGGGC-3′	292	XM_013962473.2
STAR	F 5′-AATCCACTTGGGTCTGCGAG-3′ R 5′-CACTTTCGCCACATCGAGAAC-3′	262	XM_013975437.2
CYP11	F 5′-CACTTTCGCCACATCGAGAAC-3′ R 5′-AGGCTCCTGACTTCTTAAACAGG-3′	217	NM_001287574.1
CYP17	F 5′-AACGCCATAGCAAGGAACGA-3′ R 5′-TCTGTCACGCTGTGTTGTGT-3′	263	NM_001255003.3
CYP19	F 5′-TGGGCTATGTGGACGTGTTG-3′ R 5′-TTCACCACGTTTCTCGGCAA-3′	188	NM_001123000.1
ESR	F 5′-ATGCATCCAACACCAATGGC-3′ R 5′-TCTGAGCCCCAACCCATAGA-3′	213	NM_000125.4
ESRβ	F 5′-CTGCTGGCTTTTTGGACACC-3′ R 5′-CCGGCCTTGCCTTCTCTAAA-3′	297	NM_000125.4
PGR	F 5′-CAGCCAGAGCCCACAGTACA-3′ R 5′-TGCAATCGTTTCTTCCAGCA-3′	176	XM_027979146.1
FSHR	F 5′-TGAGCAAGTGTGGCTGCTAT-3′ R 5′-ATGTGTAGAAGCACTGTCAGC-3′	320	NM_000145.4
ACTB	F 5′-GGCCATGA-3′ R 5′-CCACGCTCCGTGAGAATCTT-3′	252	NM_001314342.1

**Table 3:** Details of specific antibodies used for Western blot in the experiment.

Antibodies	Cat NO.	Source	Dilution	Observed Band (kDa)
Anti-KDM5B/PLU1/Jarid1B Antibody	Ab181089	Abcam	1:5000	170
Anti-PCNA Antibody	Ab18197	Abcam	1:500	29
BAX Rabbit Polyclonal Antibody	50599-2-Ig	ProteinTech	1:2000	24
Bcl-2(D17C4) Rabbit mAb	3498	Cell Signaling Technology	1:1000	26
Caspase 3 Rabbit Polyclonal Antibody	19677-1-AP	Abcam	1:1000	35
Caspase 9 Rabbit Polyclonal Antibody	10380-1-AP	Abcam	1:500	46,35
CDK6 Polyclonal Antibody	14052-1-AP	Proteintech	1:1000	36
Cyclin D1 Monoclonal Antibody	60186-1-IG	Proteintech	1:1000	34
Ccnd 3 Antibody	ET1612-4	Huabio	1:1000	32
MTF1 Rabbit pAb	A10824	ABclonal	1:500	122
P-Histone H2A.X	sc-517348	Santa Cruz Biotechnology	1:1000	15
Anti-beta Actin Antibody	ab8227	Abcam	1:4000	42
Alpha Tubulin Monoclonal Antibody	66031-1-Ig	Proteintech	1:10,000	52
Pierce goat anti-rabbit IGG	31460	Pierce	1:10,000	
HRP-Conjugated Affinipure Goat Anti-Mouse IgG (H + L)	SA00001-1	Proteintech	1:5000	

## Data Availability

Not applicable.

## References

[1] Solovova O.A., Chernykh V.B. (2022). Genetics of Oocyte Maturation Defects and Early Embryo Development Arrest. Genes.

[2] Nivet A.L., Leveille M.C., Leader A., Sirard M.A. (2016). Transcriptional characteristics of different sized follicles in relation to embryo transferability: Potential role of hepatocyte growth factor signalling. Mol. Hum. Reprod..

[3] Rodgers R.J., Lavranos T.C., van Wezel I.L., Irving-Rodgers H.F. (1999). Development of the ovarian follicular epithelium. Mol. Cell. Endocrinol..

[4] Safdar M., Liang A., Rajput S.A., Abbas N., Zubair M., Shaukat A., Rehman A.U., Jamil H., Guo Y., Ullah F. (2021). Orexin-A Regulates Follicular Growth, Proliferation, Cell Cycle and Apoptosis in Mouse Primary Granulosa Cells via the AKT/ERK Signaling Pathway. Molecules.

[5] Fan Y., Chang Y., Wei L., Chen J., Li J., Goldsmith S., Silber S., Liang X. (2019). Apoptosis of mural granulosa cells is increased in women with diminished ovarian reserve. J. Assist. Reprod. Genet..

[6] Zhen J., Li J., Li X., Wang X., Xiao Y., Sun Z., Yu Q. (2021). Downregulating lncRNA NEAT1 induces proliferation and represses apoptosis of ovarian granulosa cells in polycystic ovary syndrome via microRNA-381/IGF1 axis. J. Biomed. Sci..

[7] Lee J.H., Berger J.M. (2019). Cell Cycle-Dependent Control and Roles of DNA Topoisomerase II. Genes.

[8] Turrero García M., Chang Y., Arai Y., Huttner W.B. (2016). S-phase duration is the main target of cell cycle regulation in neural progenitors of developing ferret neocortex. J. Comp. Neurol..

[9] Neganova I., Lako M. (2008). G1 to S phase cell cycle transition in somatic and embryonic stem cells. J. Anat..

[10] Blackford A.N., Stucki M. (2020). How Cells Respond to DNA Breaks in Mitosis. Trends Biochem. Sci..

[11] Williams G.H., Stoeber K. (2012). The cell cycle and cancer. J. Pathol..

[12] Hume S., Dianov G.L., Ramadan K. (2020). A unified model for the G1/S cell cycle transition. Nucleic Acids Res..

[13] Yeh H.Y., Lin S.W., Wu Y.C., Chan N.L., Chi P. (2017). Functional characterization of the meiosis-specific DNA double-strand break inducing factor SPO-11 from C. elegans. Sci. Rep..

[14] Takanami T., Mori A., Takahashi H., Horiuchi S., Higashitani A. (2003). Caenorhabditis elegans Ce-rdh-1/rad-51 functions after double-strand break formation of meiotic recombination. Chromosome Res..

[15] Somfai T., Haraguchi S., Dang-Nguyen T.Q., Kaneko H., Kikuchi K. (2023). Vitrification of porcine immature oocytes and zygotes results in different levels of DNA damage which reflects developmental competence to the blastocyst stage. PLoS ONE.

[16] Pedroza-Garcia J.A., Xiang Y., De Veylder L. (2022). Cell cycle checkpoint control in response to DNA damage by environmental stresses. Plant J..

[17] Ovejero-Sánchez M., Rubio-Heras J., Vicente de la Peña M.D.C., San-Segundo L., Pérez-Losada J., González-Sarmiento R., Herrero A.B. (2022). Chloroquine-Induced DNA Damage Synergizes with Nonhomologous End Joining Inhibition to Cause Ovarian Cancer Cell Cytotoxicity. Int. J. Mol. Sci..

[18] Angelis K.J., Zaveska Drabkova L., Vagnerova R., Hola M. (2023). RAD51 and RAD51B Play Diverse Roles in the Repair of DNA Double Strand Breaks in Physcomitrium patens. Genes.

[19] Liu J.B., Zhang J.B., Yan X.M., Xie P.G., Fu Y., Fu X.H., Sun X.L., Han D.X., Li S.P., Zheng Y. (2023). DNA Double-Strand Break-Related Competitive Endogenous RNA Network of Noncoding RNA in Bovine Cumulus Cells. Genes.

[20] Ui A., Chiba N., Yasui A. (2020). Relationship among DNA double-strand break (DSB), DSB repair, and transcription prevents genome instability and cancer. Cancer Sci..

[21] Gong Y., Luo S., Fan P., Zhu H., Li Y., Huang W. (2020). Growth hormone activates PI3K/Akt signaling and inhibits ROS accumulation and apoptosis in granulosa cells of patients with polycystic ovary syndrome. Reprod. Biol. Endocrinol..

[22] Guru A., Sudhakaran G., Almutairi M.H., Almutairi B.O., Juliet A., Arockiaraj J. (2022). β-cells regeneration by WL15 of cysteine and glycine-rich protein 2 which reduces alloxan induced β-cell dysfunction and oxidative stress through phosphoenolpyruvate carboxykinase and insulin pathway in zebrafish in-vivo larval model. Mol. Biol. Rep..

[23] Forte A., Lessa P.H.C., Chaves Filho A.J.M., Aquino P.E.A., Brito L.M., Pinheiro L.C., Juruena M.F., Lucena D.F., de Rezende P.H.F., de Vasconcelos S.M.M. (2023). Oxidative stress and inflammatory process in borderline personality disorder (BPD): A narrative review. Braz. J. Med. Biol. Res..

[24] Glanzner W.G., Gutierrez K., Rissi V.B., de Macedo M.P., Lopez R., Currin L., Dicks N., Baldassarre H., Agellon L.B., Bordignon V. (2020). Histone Lysine Demethylases KDM5B and KDM5C Modulate Genome Activation and Stability in Porcine Embryos. Front. Cell Dev. Biol..

[25] Huang J., Zhang H., Wang X., Dobbs K.B., Yao J., Qin G., Whitworth K., Walters E.M., Prather R.S., Zhao J. (2015). Impairment of preimplantation porcine embryo development by histone demethylase KDM5B knockdown through disturbance of bivalent H3K4me3-H3K27me3 modifications. Biol. Reprod..

[26] Di Nisio E., Licursi V., Mannironi C., Buglioni V., Paiardini A., Robusti G., Noberini R., Bonaldi T., Negri R. (2023). A truncated and catalytically inactive isoform of KDM5B histone demethylase accumulates in breast cancer cells and regulates H3K4 tri-methylation and gene expression. Cancer Gene Ther..

[27] Lu P.J., Sundquist K., Baeckstrom D., Poulsom R., Hanby A., Meier-Ewert S., Jones T., Mitchell M., Pitha-Rowe P., Freemont P. (1999). A novel gene (PLU-1) containing highly conserved putative DNA/chromatin binding motifs is specifically up-regulated in breast cancer. J. Biol. Chem..

[28] Hayami S., Yoshimatsu M., Veerakumarasivam A., Unoki M., Iwai Y., Tsunoda T., Field H.I., Kelly J.D., Neal D.E., Yamaue H. (2010). Overexpression of the JmjC histone demethylase KDM5B in human carcinogenesis: Involvement in the proliferation of cancer cells through the E2F/RB pathway. Mol. Cancer.

[29] Wang D., Han S., Peng R., Jiao C., Wang X., Yang X., Yang R., Li X. (2016). Depletion of histone demethylase KDM5B inhibits cell proliferation of hepatocellular carcinoma by regulation of cell cycle checkpoint proteins p15 and p27. J. Exp. Clin. Cancer Res..

[30] Barrett A., Madsen B., Copier J., Lu P.J., Cooper L., Scibetta A.G., Burchell J., Taylor-Papadimitriou J. (2002). PLU-1 nuclear protein, which is upregulated in breast cancer, shows restricted expression in normal human adult tissues: A new cancer/testis antigen?. Int. J. Cancer.

[31] McCann C., Quinteros M., Adelugba I., Morgada M.N., Castelblanco A.R., Davis E.J., Lanzirotti A., Hainer S.J., Vila A.J., Navea J.G. (2022). The mitochondrial Cu(+) transporter PiC2 (SLC25A3) is a target of MTF1 and contributes to the development of skeletal muscle in vitro. Front. Mol. Biosci..

[32] Bi S.S., Talukder M., Jin H.T., Lv M.W., Ge J., Zhang C., Li J.L. (2022). Nano-selenium alleviates cadmium-induced cerebellar injury by activating metal regulatory transcription factor 1 mediated metal response. Anim. Nutr..

[33] Deng M., Wan Y., Chen B., Dai X., Liu Z., Yang Y., Cai Y., Zhang Y., Wang F. (2021). Long non-coding RNA lnc_3712 impedes nuclear reprogramming via repressing Kdm5b. Mol. Ther. Nucleic Acids.

[34] Sedelnikova O.A., Rogakou E.P., Panyutin I.G., Bonner W.M. (2002). Quantitative detection of (125)IdU-induced DNA double-strand breaks with gamma-H2AX antibody. Radiat. Res..

[35] Słupianek A., Pytel D., Majsterek I. (2007). The role of oncogenic tyrosine kinases in the cellular response to anticancer therapy. Postepy Hig. Med. Dosw..

[36] Shahin W.S., Ebed S.O., Tyler S.R., Miljkovic B., Choi S.H., Zhang Y., Zhou W., Evans I.A., Yeaman C., Engelhardt J.F. (2023). Redox-dependent Igfbp2 signaling controls Brca1 DNA damage response to govern neural stem cell fate. Nat. Commun..

[37] Ovejero-Sanchez M., Gonzalez-Sarmiento R., Herrero A.B. (2021). Synergistic effect of Chloroquine and Panobinostat in ovarian cancer through induction of DNA damage and inhibition of DNA repair. Neoplasia.

[38] Srinivas U.S., Tan B.W.Q., Vellayappan B.A., Jeyasekharan A.D. (2019). ROS and the DNA damage response in cancer. Redox Biol..

[39] Hübner C., Haase H. (2021). Interactions of zinc- and redox-signaling pathways. Redox Biol..

[40] Huang S., Cao B., Zhang J., Feng Y., Wang L., Chen X., Su H., Liao S., Liu J., Yan J. (2021). Induction of ferroptosis in human nasopharyngeal cancer cells by cucurbitacin B: Molecular mechanism and therapeutic potential. Cell Death Dis..

[41] Li J., Wan X., Qiang W., Li T., Huang W., Huang S., Wu D., Li Y. (2015). MiR-29a suppresses prostate cell proliferation and induces apoptosis via KDM5B protein regulation. Int. J. Clin. Exp. Med..

[42] Yoo J., Kim G.W., Jeon Y.H., Kim J.Y., Lee S.W., Kwon S.H. (2022). Drawing a line between histone demethylase KDM5A and KDM5B: Their roles in development and tumorigenesis. Exp. Mol. Med..

[43] Cui G., Liu D., Li W., Li Y., Liang Y., Shi W., Zhao S. (2017). Original Research: miR-194 inhibits proliferation and invasion and promotes apoptosis by targeting KDM5B in esophageal squamous cell carcinoma cells. Exp. Biol. Med..

[44] Yang Y., Li W., Wei B., Wu K., Liu D., Zhu D., Zhang C., Wen F., Fan Y., Zhao S. (2020). MicroRNA let-7i Inhibits Histone Lysine Demethylase KDM5B to Halt Esophageal Cancer Progression. Mol. Ther. Nucleic Acids.

[45] Scully R., Panday A., Elango R., Willis N.A. (2019). DNA double-strand break repair-pathway choice in somatic mammalian cells. Nat. Rev. Mol. Cell Biol..

[46] Tarsounas M., Sung P. (2020). The antitumorigenic roles of BRCA1-BARD1 in DNA repair and replication. Nat. Rev. Mol. Cell Biol..

[47] Li X., Heyer W.D. (2008). Homologous recombination in DNA repair and DNA damage tolerance. Cell Res..

[48] Awwad S.W., Darawshe M.M., Machour F.E., Arman I., Ayoub N. (2023). Recruitment of RBM6 to DNA Double-Strand Breaks Fosters Homologous Recombination Repair. Mol. Cell. Biol..

[49] Imamura R., Saito M., Shimada M., Kobayashi J., Ishiai M., Matsumoto Y. (2023). APTX acts in DNA double-strand break repair in a manner distinct from XRCC4. J. Radiat. Res..

[50] Gale M., Sayegh J., Cao J., Norcia M., Gareiss P., Hoyer D., Merkel J.S., Yan Q. (2016). Screen-identified selective inhibitor of lysine demethylase 5A blocks cancer cell growth and drug resistance. Oncotarget.

[51] Bonacci T., Emanuele M.J. (2020). Dissenting degradation: Deubiquitinases in cell cycle and cancer. Semin. Cancer Biol..

[52] Wang Z., Tang F., Qi G., Yuan S., Zhang G., Tang B., He S. (2015). KDM5B is overexpressed in gastric cancer and is required for gastric cancer cell proliferation and metastasis. Am. J. Cancer Res..

